# Correction to: Longitudinal neuromelanin changes in prodromal and early Parkinson's disease in humans and rat model

**DOI:** 10.1093/braincomms/fcag030

**Published:** 2026-02-12

This is a correction to: Jean-Baptiste Pérot, Anthony Ruze, Rahul Gaurav, Sana Rebbah, Capucine Cadin, Arnaud Le Troter, Lucas Soustelle, Laura Mouton, Romain Valabrègue, Annabelle Parent, Graziella Mangone, François-Xavier Lejeune, Isabelle Arnulf, Jean-Christophe Corvol, Marie Vidailhet, Mathieu D Santin, Miquel Vila, Stéphane Lehéricy, Longitudinal neuromelanin changes in prodromal and early Parkinson's disease in humans and rat model, *Brain Communications*, Volume 7, Issue 3, 2025, https://doi.org/10.1093/braincomms/fcaf204

In the originally published version of the manuscript, there was an error in Figure 4. The brain image labelled “R1” actually displays a T1 contrast. The error does not affect the quantitative results shown in Figure 4D, which were derived from R1 maps.

Figure 4 should read:

**Figure:**
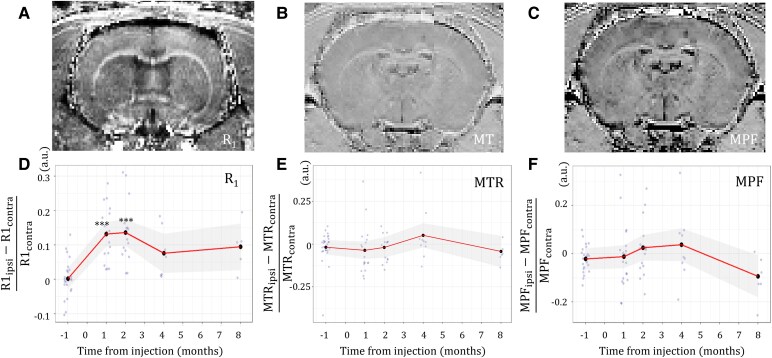


instead of:

**Figure:**
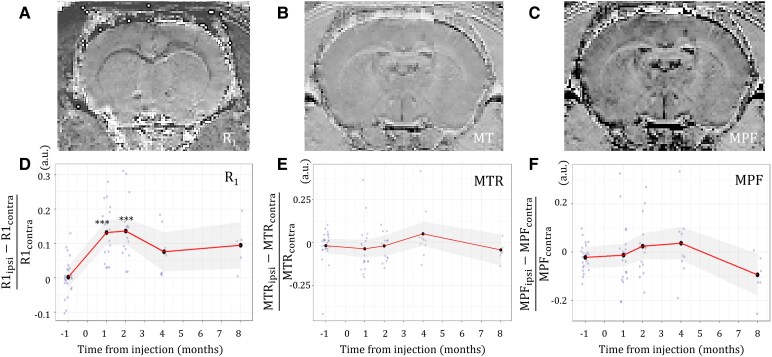


The authors apologize for the mistake. The emendation has been made to the article.

