# Supplementary material for: Longitudinal neuromelanin changes in prodromal and early Parkinson’s disease in humans and rat model
Source: Brain Commun. 2025 May 28;7(3):fcaf204. doi: 10.1093/braincomms/fcaf204 (PMC12149847; doi:10.1093/braincomms/fcaf204)
Supplement: fcaf204_Supplementary_Data [file fcaf204_supplementary_data.zip › Supplementary Information.pdf]

Supplementary Information

Surgical procedures

Anesthesia was induced by inhalation of 3% isoflurane and maintained with 1.5-2% isoflurane. Rats received subcutaneous injection of Buprenorphine at 0.1mg/kg for analgesia 20 min prior to injection. Then skin and subcutaneous tissue were incised in stereotaxic frame, and skull was perforated 5.2 mm posterior and 2 mm right to the bregma. After perforation, 2µL of AAV-hTyr solution was injected 7.6 mm below the dura mater at a speed of 0.4 µL/min. After injection the subcutaneous tissue and the skin were sutured separately with resorbing thread. The surgical procedure lasted 1h30. Body temperature of the rats was monitored and regulated during the whole procedure. A second injection of Buprenorphine was realized 4-6 hours after the injection. Buprenorphine was mixed to the drinking water for 24h.

Preclinical MRI protocol

Preclinical MRI protocol included 2D T_1 weighted image for neuromelanin imaging (FLASH, TR/TE=333/6.8ms, in-plane resolution=150x150μm^2, 16 slices, slice thickness=0.4mm, acquisition time=12 min) ; three 3D multi gradient echo sequences with varying flip angles (FA) and MT preparation were acquired for quantitative MRI (TR=47ms, TE_1:ΔTE:TE_L=1.71:1.91:30.36ms, 16 echoes, spatial resolution=200µm isotropic, whole brain coverage, t_{MT}=10ms, $B_{1,MTe}^{peak}$=7.8µT, acquisition time=32 min each). The specific parameters for the three sequences were respectively FA=6°, offset frequency (Δ) of 100kHz (MTOFF_FA6); FA=24°, Δ=100kHz (MTOFF_FA24); FA=6°, Δ=5kHz (MTON_FA6) ; and a 2D Multi Spin Echo sequence for T_2 mapping (TR/TE=4000/7ms, 8 echoes, in-plane resolution=150x150 μm^2, slice thickness=400 µm, 50 slices, acquisition time=12min48s, n=12 animals at baseline and 1mpi, n=6 at 2mpi and 4mpi, and n=5 at 8mpi).

Quantitative Susceptibility Mapping

For QSM generation procedure, raw data were exported, and complex images (magnitude and phase) were then reconstructed with in-house software in Matlab (Mathworks, USA). The signal magnitude and phase from multiple channels of the receive coil were combined using an in-house optimized method. Then, the local field map was calculated by non-linear fitting of the complex gradient echo signal over echo times(Liu et al., 2013), background magnetic fields were removed using the Laplacian boundary value method(Zhou et al., 2014) and the local

field-to-magnetic susceptibility inverse problem was solved using the L1-Morphology Enabled Dipole Inversion method(de Rochefort et al., 2008).

Quantitative Magnetization Transfer

Macromolecular Proton Fraction (MPF) was calculated from MTOFF_FA6, MTON_FA6, T_1 and B_1 maps using Yarnykh's single-point method(Yarnykh, 2012) with optimized parameters at 11.7T(Naumova et al., 2017). Python code for MPF estimation can be found at https://github.com/lsoustelle/qMT.

Immunohistochemistry

Animals were anesthetized with inhalation of isoflurane and injected intraperitoneally with xylazine (500mg/kg) for analgesia. After 15 minutes rats received a lethal dose of Euthasol (500mg/kg) intraperitoneally. After death, perfusion of PBS was performed in the left cardiac ventricle for 6 min, followed with perfusion of 4% paraformaldehyde (PFA). Brain was then extracted and post-fixed for 3 days in 4% PFA. Brains were then washed 3 times with PBS and plunged in a solution of 30% sucrose for cryoprotection. After sinking, brains were frozen in -50°C isopentane and stored at -80°C. Brains were then sliced at -20°C with a cryostat. SN was sampled with 2 series of 10 microscope slides, each containing 6 brain slices 14 µm thick. Slides were stored at -80°C. Slides were dried at room temperature for 30 min and rinsed 2 times in wash buffer. Samples were incubated in Blocker/diluent solution for 10 min and rinsed 2 times in wash buffer. Slides were then incubated overnight at 4°C with primary antibody (1:1000, Calbiochem, Cat#657012) and rinsed 5 min in wash buffer before 30 min incubation with Multiview Plus Rabbit AP secondary antibody solution. After 2 rinses in wash buffer the staining was revealed in anti-rabbit blue AP Chromogen substrate for 10 min at room temperature. Following revelation, samples were rinsed 3 times in wash buffer and 2 times in distilled water and then mounted in Mowiol 4-88 aqueous solution. Quantification of intracellular neuromelanin was realized with slides mounted in Mowiol 4-88 aqueous solution directly after unfreezing and 3 rinses in distilled water, with no staining.

Intracellular NM quantification

Intracellular NM density was quantified by measuring optical density of intracellular NM in unstained slices. After drying of mounting medium, slides were scanned using an Olympus Slideview VS200 slide scanner together with the Olyvia 3.3 software to obtain 20x high

resolution micrographs of the samples. For each animal, a minimum of 30 cells were sampled in the entire ipsilateral substantia nigra. NM was manually delineated in cells by two different raters blinded to the time of euthanasia. Mean optical density of NM was extracted for each cell and normalized by optical density of the background. Then, mean NM optical density was calculated for each animal by averaging the normalized NM optical density values of all cells.

Clinical MRI protocol

MRI was performed for all subjects on a Siemens Prisma 3T scanner (Siemens Healthineers) with a 64-channel head reception coil. Whole brain anatomic image was acquired with T_1-weighted 3D MP2RAGE (TR/TE=5000/2.98 ms, voxel size=1x1x1 mm^3, acquisition time=8 min 12 s). NM-sensitive MRI was acquired using T_1-weighted 2D turbo spin echo (TR/TE=890/13 ms, in-plane resolution=0.3x0.3 mm^2, 48 slices, slice thickness=3 mm, acquisition time=6 min 55 s).

Clinical examination

Clinical examination included the Hoehn and Yahr scale, which evaluates the severity of Parkinson's disease-related symptoms(Hoehn & Yahr, 1967); the Movement Disorder Society Unified Parkinson's Disease Rating Scale (MDS-UPDRS) part III in the OFF condition, which evaluates motor disability >12 hours after withdrawing dopaminergic treatment(Goetz et al., 2008); and the Mattis dementia rating scale (MDRS(Mattis, 1988)) and the Montréal cognitive assessment score (MoCA(Nasreddine et al., 2005)) , which evaluate cognitive impairment.

Statistical Analyses

Longitudinal data were analyzed by fitting separate Linear Mixed Models (LMMs) to each imaging modality using the "*lmer*" function in the lme4 package (v1.1-31)(Bates et al., 2015). All LMMs included time from injection as independent variable, with a random intercept to account for the repeated measurements per animal. To compare histological measurements over time, which consisted in independent observation points between the time points, we performed one-way analysis of variance (ANOVA) with time from injection as a factor. Significance of all the main effects or the interaction effects were assessed by Type II Wald chisquare tests using the "*Anova*" function in the car package (v3.1-1). Post-hoc pairwise comparisons were then performed on the significant effect based on estimated marginal means from the linear models using the "*emmeans*" function in the emmeans package (V.4.5). The resulting p-values of the

pairwise comparisons were adjusted for multiple comparisons using the false discovery rate (FDR) method. Additionally, to examine the effect of laterality, we tested the estimated means of the change rates against zero at each timepoint.

Correlations between the changes in NM-MRI and histological quantification were examined using Spearman's rank correlation coefficients (Spearman's rho). Longitudinal association between the MR parameters was analyzed using repeated measures correlations with the rmcorr package (v0.6.0)(Bakdash & Marusich, 2017).

To investigate the age-evolution of the NM signal in the subjects of the ICEBERG cohort, the NM signal data were normalized by dividing all values by the mean value of the HV group calculated at baseline (110.9) and multiplying by 100. For the sake of visualization and comparison, the PD patients' baseline ages were aligned arbitrarily to the mean age at onset of the PD group. Doing this allowed to consider and compare patients by disease duration rather than their less meaningful actual age. An approximation of the evolution of the NM signal was made graphically on the basis of the cohort follow-up data by fitting the data of each group with a quadratic trend (HV and iRBD) and a linear trend (PD and an isolated fourth group of 7 iRBDs that converted to PD during the follow-up) using the "*geom_smooth*" function of the ggplot2 package (v3.4.2) (Wickham, 2016, http://ggplot2.org). The degree of decrease in the NM signal for both PD and converted iRBD groups was reported with the slope (r) of the linear regression line and its standard error (SE), and then compared with each other using the "*emtrends*" function in the emmeans package.

Supplementary bibliography

Bakdash, J. Z., & Marusich, L. R. (2017). Repeated Measures Correlation. *Frontiers in Psychology*, *8*. https://www.frontiersin.org/articles/10.3389/fpsyg.2017.00456

Bates, D., Mächler, M., Bolker, B., & Walker, S. (2015). Fitting Linear Mixed-Effects Models Using lme4. *Journal of Statistical Software*, *67*, 1-48. https://doi.org/10.18637/jss.v067.i01

de Rochefort, L., Brown, R., Prince, M. R., & Wang, Y. (2008). Quantitative MR susceptibility mapping using piece-wise constant regularized inversion of the magnetic field. *Magnetic Resonance in Medicine*, *60*(4), 1003-1009. https://doi.org/10.1002/mrm.21710

Goetz, C. G., Tilley, B. C., Shaftman, S. R., Stebbins, G. T., Fahn, S., Martinez-Martin, P., Poewe, W., Sampaio, C., Stern, M. B., Dodel, R., Dubois, B., Holloway, R., Jankovic, J., Kulisevsky, J.,

Lang, A. E., Lees, A., Leurgans, S., LeWitt, P. A., Nyenhuis, D., … LaPelle, N. (2008). Movement Disorder Society-sponsored revision of the Unified Parkinson's Disease Rating Scale (MDS-UPDRS) : Scale presentation and clinimetric testing results. *Movement Disorders*, *23*(15), 2129-2170. https://doi.org/10.1002/mds.22340

Hoehn, M. M., & Yahr, M. D. (1967). Parkinsonism. *Neurology*, *17*(5), 427-427. https://doi.org/10.1212/WNL.17.5.427

Liu, T., Wisnieff, C., Lou, M., Chen, W., Spincemaille, P., & Wang, Y. (2013). Nonlinear formulation of the magnetic field to source relationship for robust quantitative susceptibility mapping. *Magnetic Resonance in Medicine*, *69*(2), 467-476. https://doi.org/10.1002/mrm.24272

Mattis, S. (1988). *Dementia rating scale : DRS : professional manual*. PAR.

Nasreddine, Z. S., Phillips, N. A., Bédirian, V., Charbonneau, S., Whitehead, V., Collin, I., Cummings, J. L., & Chertkow, H. (2005). The Montreal Cognitive Assessment, MoCA : A brief screening tool for mild cognitive impairment. *Journal of the American Geriatrics Society*, *53*(4), 695-699. https://doi.org/10.1111/j.1532-5415.2005.53221.x

Naumova, A. V., Akulov, A. E., Khodanovich, M. Yu., & Yarnykh, V. L. (2017). High-resolution three-dimensional macromolecular proton fraction mapping for quantitative neuroanatomical imaging of the rodent brain in ultra-high magnetic fields. *NeuroImage*, *147*, 985-993. https://doi.org/10.1016/j.neuroimage.2016.09.036

Wickham, H. (2016). *ggplot2 : Elegant Graphics for Data Analysis*. Springer International Publishing.

Yarnykh, V. L. (2012). Fast macromolecular proton fraction mapping from a single off-resonance magnetization transfer measurement. *Magnetic Resonance in Medicine*, *68*(1), 166-178. https://doi.org/10.1002/mrm.23224

Zhou, D., Liu, T., Spincemaille, P., & Wang, Y. (2014). Background field removal by solving the Laplacian boundary value problem. *NMR in Biomedicine*, *27*(3), 312-319. https://doi.org/10.1002/nbm.3064
